# Variations existing in protein-coding genes of the complete mitochondrial genomes of two highly morphological similarity fish: *Periophthalmus magnuspinnatus* and *P. modestus* (Perciformes, Gobiidae)

**DOI:** 10.1080/23802359.2016.1167638

**Published:** 2016-08-21

**Authors:** Dan Wang, Yanmei Liu, Qiang Li, Lei Wang, Zhizhi Liu

**Affiliations:** a Key Laboratory of Exploration and Utilization of Aquatic Genetic Resources, Ministry of Education, Shanghai Ocean University, Shanghai, China;; b Central Laboratory of Shanghai Xuhui Central Hospital, Shanghai, China

**Keywords:** Gobiidae, mitochondrial, *Periophthalmus magnuspinnatus*, *Periophthalmus modestus*, phylogenetic analysis

## Abstract

*Periophthalmus magnuspinnatus* is a new record species of Gobiidae in China. It had been misidentified as *P. modestus* for long time early before 2006. Here, the complete mitochondrial genome (mtDNA) sequences of the two species were first reported and analyzed comparably. The two genomes were both consisted of 13 protein-coding genes, 22 tRNA genes, two rRNA genes and a control region. Except for eight tRNA and ND6 genes, all other mitochondrial genes were encoded on the heavy strand. It was 16,496 bp and 16,803 bp, respectively, and *P. modestus* had a 238 bp-gap between tRNAleu and ND5. There were high variations (10–19%) in the protein-coding genes. In the initiation condon and stop codons, the two fish also had tiny difference. Phylogenetic analysis showed that *P. magnuspinnatus* and *P. modestus* first clustered together and then they constituted Gobiidae clade with other 12 fish. Whereas Odontobutidae and Rhyacichthyidae formed the sister group, then clustered with Eleotridae, and finally joined with Gobiidae, which is consist with previous phenotypic report. The study will contribute to the phylogenetic analysis of the Gobiidae and natural resources conservation of *P. magnuspinnatus* and *P. modestus*.

Mudskippers are the largest amphibious teleost fishes and they are more and more attracting attentions of environmental experts and the public. The mudskipper *Periophthalmus magnuspinnatus* (Perciformes, Gobiidae) was first described in Korea by Lee et al. (1995) and now is a new record species (Wang et al. 2006) in China. In fact, it is a common fish in the coastal waters from Bohai Sea to South China Sea. However, before 2006, the fish had been misidentified as another mudskipper *P. modestus* for their highly morphological similarity (Wang et al. 2006). In 2006, researchers found that the similarities of some sequences of *Cyt b* gene in P. modestus varied more than 15 percent (Wang et al. 2006). This results absorbed well-known fish taxonomy professor Wu’s concerns. Then, under his guide of careful identification by morphological characters, researchers finally confirmed the new record species and solved Lee’s doubts and suspicions (Lee et al. 1995). However, we still wanted to know how large difference existed in the rest genes of mtDNA for the two fish and which genes should be better for the phylogenetic analysis of Gobiidae?

In 2015, about 30 natural samples were collected randomly from Jiuduansha wetland in Yangtz River estuary (31°03′˜31°17′N, 121°46′–122°15′E). The samples were stored in 75% ethanol at 4 °C in Fish Herbarium, Shanghai Ocean University, Shanghai. The genomic DNA was extracted from pectoral fin using phenol/chloroform procedure (Sambrook & Russell, 2001). Primers were designed according to *Boleophthalmus pectinirostri*s (Liu et al. 2012). Then we used DNA baser V.3.5.4 to assemble the whole sequence. The assembled mitochondrial genome was annotated by Mitoannotator and Mitofish (Iwasaki et al. 2013). All transfer RNA genes were reappraised by tRNA Scan-SE version 1.21 (Lowe & Eddy, 1997). To detect tandem repeat as existed in the control region of B. pectinirostris (Liu et al. 2012), we examined ten individuals for *P. magnuspinnatus* and *P. modestus*, respectively. In order to analyse the phylogenetic relationships, we downloaded 23 mtDNA of relative species. These sequences were aligned by Clustal X with default settings (Thompson et al. 1997). The best-fit model to sequence evolution was selected in TN93 + G + I by Akaire information criteria (AICc). Finally, the maximum-likelihood (ML) phylogenetic tree was conducted by MEGA 6.0 (Koichiro et al. 2013), with 1000 bootstrap replicates.

The two new mtDNA of *P. modestus* (KP 638476) and *P. magnuspinnatus* (KT 357639) were 16,803 bp and 16,496 bp in length, respectively. A 238 bp-gap between *tRNAleu* and *ND5* existed in the mtDNA of *P. modestus*. The two mtDNA were both consisted of 13 typical vertebrate protein-coding genes, 22 tRNAs, two rRNAs and a control region. All genes’ arrangement was identical to other gobies. Except for eight tRNA and *ND6* genes, all other mitochondrial genes were encoded on the heavy strand. By comparison, we detected relatively high variations (10–19%) in the 13 protein-coding genes of the two mudskippers. However, apart from tRNAAsp (11%) and tRNATrp (8%), the rest 22 genes’ variation was only 0–7%. This result was in accordance with Wang’s report (Wang et al. 2006). Again, we believe that molecular analysis of suitable genes will sometimes promote traditional taxonomy of fishes. As to start codon, there was slight deference between *P. magnuspinnatus* and *P. modestus*. In *P. modetus*, ATP6 used CTG. Whereas *COI* selected GTG as start codon in the two fish. Besides these, the rest 11 or 12 protein-coding genes initiated with ATG. Similarly, there was tiny difference in the stop codons. *Periophthalmus magnuspinnatus* used TAG, TAA and T, but *P. modestus* preferred TAA, TA and T. The content A + T was 55.6% and 55.4% for *P. modestus* and *P. magnuspinnatus*, respectively. The phenomenon of A + T content higher than G + C content was similar to those of *B. pectinirostris* (Liu et al. 2012) and *Odontobutis potamophila* (Li & Liu 2016). Analysis showed that *ND4* and *ND5* had the largest number of mutation loci, indicating that they would be better molecular markers than *Cyt b* gene in examining intraspecific population genetic diversity such as *P. magnuspinnatus* and *P. modestus*. We did not detect any tandem repeats in the two mudskippers, and this is unlike *B. pectinirostris* (Liu et al. 2012), *O. platycephala* and *O. sinensis* (Ma et al. 2015).

In the phylogenetic analysis, *P. magnuspinnatus* and *P. modestus* first clustered together (Figure 1). Then, they constituted Gobiidae clade with other 12 fish. Whereas the seven fish in Odontobutidae or Rhyacichthyidae formed the sister group, then clustered with Eleotridae, and finally joined with Gobiidae (Figure 1). The relationship is accordant with previous phenotypic report (Li et al. 2015; Zang et al. 2016). The study will contribute (Figure 1) to the phylogenetic analysis of the Gobiidae and natural resources conservation of *P. magnuspinnatus* and *P. modestus*.

**Figure 1.**
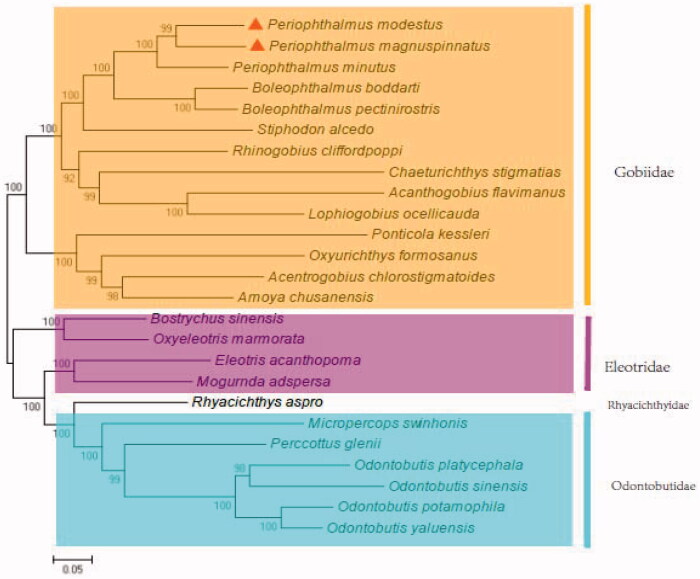
The phylogenetic relationship for fish of the Gobioidei.
